# *Achilles*, a New Family of Transcriptionally Active Retrotransposons from the Olive Fruit Fly, with Y Chromosome Preferential Distribution

**DOI:** 10.1371/journal.pone.0137050

**Published:** 2015-09-23

**Authors:** Konstantina T. Tsoumani, Elena Drosopoulou, Kostas Bourtzis, Aggeliki Gariou-Papalexiou, Penelope Mavragani-Tsipidou, Antigone Zacharopoulou, Kostas D. Mathiopoulos

**Affiliations:** 1 Department of Biochemistry and Biotechnology, University of Thessaly, Larissa, Greece; 2 Department of Genetics, Development and Molecular Biology, Aristotle University of Thessaloniki (AUTH), Thessaloniki, Greece; 3 Insect Molecular Genetics Group, IMBB, Vassilika Vouton, 71110 Heraklion, Crete, PO Box 1527, Greece; 4 Department of Environmental and Natural Resources Management, University of Patras, Agrinio, Greece; 5 Insect Pest Control Laboratory, Joint FAO/IAEA Division of Nuclear Techniques in Food and Agriculture, Vienna, Austria; 6 Department of Biology, Division of Genetics, Cell and Developmental Biology, University of Patras, Patras, Greece; University of Helsinki, FINLAND

## Abstract

Sex chromosomes have many unusual features relative to autosomes. The in depth exploration of their structure will improve our understanding of their origin and divergence (degeneration) as well as the evolution of genetic sex determination pathways which, most often are attributed to them. In Tephritids, the structure of Y chromosome, where the male-determining factor M is localized, is largely unexplored and limited data concerning its sequence content and evolution are available. In order to get insight into the structure and organization of the Y chromosome of the major olive insect pest, the olive fly *Bactrocera oleae*, we characterized sequences from a Pulse Field Gel Electrophoresis (PFGE)-isolated Y chromosome. Here, we report the discovery of the first olive fly LTR retrotransposon with increased presence on the Y chromosome. The element belongs to the *BEL-Pao* superfamily, however, its sequence comparison with the other members of the superfamily suggests that it constitutes a new family that we termed *Achilles*. Its ~7.5 kb sequence consists of the 5’LTR, the 5’non-coding sequence and the open reading frame (ORF), which encodes the polyprotein Gag-Pol. *In situ* hybridization to the *B*. *oleae* polytene chromosomes showed that *Achilles* is distributed in discrete bands dispersed on all five autosomes, in all centromeric regions and in the granular heterochromatic network corresponding to the mitotic sex chromosomes. The between sexes comparison revealed a variation in *Achilles* copy number, with male flies possessing 5–10 copies more than female (CI range: 18–38 and 12–33 copies respectively per genome). The examination of its transcriptional activity demonstrated the presence of at least one intact active copy in the genome, showing a differential level of expression between sexes as well as during embryonic development. The higher expression was detected in male germline tissues (testes). Moreover, the presence of *Achilles*-like elements in different species of the Tephritidae family suggests an ancient origin of this element.

## Introduction

Sex chromosomes have many unusual features compared to autosomes and since long have been the subject of genetic, cytological and recently molecular research. Sex chromosomes are implicated in multiple biological processes involving sex determination, epigenetic chromosome-wide regulation of gene expression, distribution of genes on chromosomes, genomic conflict, local adaptation, speciation and, not least, genome evolution (for review see [1]).

Current evolutionary models suggest that sex chromosomes originated from an ordinary pair of autosomes after one of them acquired a male-determining factor on a proto-Y. Massive gene loss on the proto-Y would follow, generating a typical Y chromosome in which the few remaining genes were mostly shared with the X [2–4]. The suppressed recombination between nascent X and Y chromosome, matched with male-limited transmission, led to the degeneration of the Y chromosome. This process occurred as a result of the insertion of transposable elements and other non-coding sequences on the Y, chromosomal rearrangements, accumulation of sexually antagonistic mutations and silencing of all or most of the genes present on the proto-Y [2,5].

Although this theory is very well-supported in mammals and other groups (e.g., [6]), different theories have been proposed for the evolution of dipteran Y chromosomes, suggesting that they are either degenerated Xs or neo-Ys or of a non-canonical origin [7]. Comparative studies in 37 dipteran species uncovered a tremendous hidden variation in dipteran sex chromosomes suggesting a dynamic process in which the transition between autosomes and sex chromosomes is highly labile [8,9]. Besides such fundamental differences, Y chromosomes do share several common features. Firstly, the presence of a male-determining factor in several dipterans that may have a common origin and may show a high level of sequence conservation. Secondly, they appear strongly heterochromatic, gene-poor and plentiful of highly repetitive DNA. This last set of characteristics, common to most animal Ys, make them intractable to current sequencing technologies [10,11].

Y chromosomes are also considered a sink of repetitive sequences, such as transposable elements (TEs) which are known to make up a significant proportion of the genomes of all organisms and play an important role in their evolution [12–14]. About 77% of the *D*. *melanogaster* annotated heterochromatin is occupied by fragmented and nested TEs and other repetitive DNAs [15] and in spite of their low copy number they are responsible for more than 50% of naturally-occurring mutations with major morphological effects [16]. The vast majority of TE’s correspond to retrotransposons, which include mobile elements that transpose through an RNA intermediate. LTR-retrotransposons generally contain one or two segments of coding region that are homologous to the *gag* and *pol* regions of retroviruses. Within the *pol* region are short stretches of highly conserved amino acids [17] associated with the Gag/Pol protease reverse transcriptase, RNase H or integrase domains.True LTR retrotransposons (excluding infectious and non-infectious retroviruses) can be further divided into three superfamilies based on their sequence similarity and various structural features: *Copia*, *Gypsy* and *BEL/Pao* [18] *Copia* and *Gypsy*-like elements are present in almost all eukaryotic genomes. In contrast, the *BEL/Pao* superfamily contains fewer elements that have been identified only recently [19]. While elements from the *Copia* and *Gypsy* superfamilies are widespread in eukaryotic genomes, *BEL/Pao* elements are only present in metazoan genomes, suggesting a later appearance in eukaryote evolution or a recent loss in several major eukaryotic lineages [20].

Transposable elements can directly affect gene expression by inserting either into protein-coding genes or their regulatory elements. Alternatively, transposable elements can induce large-scale chromatin structural changes (i.e., heterochromatinization of chromosomal regions) which can result in the simultaneous silencing of a large number of genes [21–23]. Thus large-scale structural changes during evolution of Y chromosomes could be attributed to some extent to the accumulation of repetitive sequences and transposable elements. Indeed, a lot of reports from a broad range of taxa suggest the TE-accumulation on sex chromosomes. A high percentage of *D*. *melanogaster* Y chromosome comprises of retrotransposons [10,24]. Similarly, a massive accumulation of retrotransposons was revealed in the neo-Y chromosomal regions of *D*. *miranda* [25–27]. Noticeably, *mtanga*, an LTR retrotransposon that is distributed in clusters and is actively expressed on the Y chromosome, has been also characterized in the malaria mosquito *Anopheles gambiae* [28]. Further molecular analysis of Y chromosome-derived sequences of this species provided evidence supporting the enrichment of TEs along this chromosomal region [29].

Except from the well-studied *D*. *melanogaster*, where most of the information is generated and most of the theories are built, Y chromosome structure and evolution in Tephritids is largely unexplored. Tephritidae is a very large family of fruit flies of major economic importance in agriculture [30]. Cytogenetic analyses of several Tephritid species have revealed the existence of six pairs of chromosomes in their metaphase karyotype, including a pair of largely heterochromatic sex chromosomes, with the male being the heterogametic sex (detailed review in [31]). Sex chromosomes remain under-replicated (albeit transcriptionally active [32] and tend to form a granular heterochromatic network in polytene spreads [33,34]. Relative comparisons of chromosome length in various Tephritids have demonstrated a considerable size variability of the Y chromosome which, in many cases, is a very small, dot-like chromosome (e.g., *Bactrocera oleae*, *B*. *dorsalis*, *B*. *tryoni*, *Anastrepha ludens* and *A*. *obliqua*). The small size of these Y chromosomes offers an opportunity to quantitatively separate them in a Pulsed Field Gel Electrophoresis (PFGE) and subsequently analyse their sequence.

In terms of sequence content of the Y chromosome of Tephritids very little is known. In *Ceratitis capitata*, the best studied member of the Tephritidae family, only some Y-specific and Y-enriched repetitive sequences have been isolated [35–37], supporting the repetitive and degenerate structure of its Y chromosome. Analysis of a series of Y chromosome deletions in male translocation lines of this species defined also the male-determining region on the long arm of the Y chromosome [38]. Clearly, these fully viable and fertile mutants maintain their maleness factor in their truncated Y chromosome, being depleted from ‘unnecessary’ sequences. A few more repetitive sequences have also been isolated and analyzed in *B*.*oleae* [34,39].

In order to get insight into the structure and organization of the Y chromosome of the major olive insect pest, the olive fly *B*.*oleae*, we isolated sequences from a PFGE-isolate Y chromosome. Here, we report the characterization of the first olive fly LTR retrotransposon that defines a new BEL/Pao family with increased presence on the Y chromosome. We termed this new *B*. *oleae* retrotransposon family *Achilles*, after the greatest ancient Greek warrior of the Trojan War.

## Materials and Methods

### Ethics statement

The study was carried out on laboratory reared olive flies. No specific permissions are required for these experiments, since these studies did not involve endangered or protected species.

### Flies and DNA preparation

The source of *B*. *oleae* was the ‘Demokritos’ strain, that is maintained in our laboratory. *B*. *oleae* genomic DNA was extracted from adult flies using the Wizard Genomic DNA extraction kit (Promega, Madison, WI, USA) and quantified spectrophotometrically.

### Isolation of Y-enriched sequences

#### Pulse field gel electrophoresis

About 2000 *B*. *oleae* eggs were collected 24–48 hours after oviposition. Eggs were dechorionated in 50% bleach solution (50% Clorox; final concentration: 2.5% hypochlorite) with gentle agitation. After about one minute, the bleach solution was drawn off and eggs were washed three times in distilled water, once in Hank’s balanced salts solution (HBSS) and resuspended in 1 ml HBSS. Eggs were crushed in a 2-ml Kontes Dounce tissue grinder and an equal volume (1 ml) of 1% pulsed field certified agarose (Bio Rad) melted in HBSS was added, quickly mixed and dispensed in PFGE plug mold. Agarose plugs were solidified at 4°C for 20 minutes and then lysed in 20 ml ESP (0.1M EDTA, 1% SDS and 50μg/ml proteinase K) at 50°C for 48 hours with a lysis solution change after ~24 hours. Plugs were finally washed and maintained in 0.5M EDTA.

Egg-containing agarose plugs were washed in 1xTBE solution prior to PFGE electrophoresis. One quarter to a full plug was used for electrophoresis. Genomic DNA was separated in a CHEF DR II apparatus (BioRad) under the same conditions used for the separation of Drosophila’s chromosome 4 [40], i.e., 0.7% agarose in 1xTBE at 50V for 180 hours at 15°C with a linearly ramped switch time from 2500 to 4500 sec. *S*. *pombe* chromosomes were used as size markers. In addition to a high molecular zone near the electrophoresis wells, an extra smeary zone was observed in the area of 3–5 Mb. This area was excised from the gel and the agarose was removed with the use of agarase. DNA was concentrated by ethanol precipitation. Initially, this material was used in *in situ* hybridization in order to verify its origin. Subsequently, it was used for preparation of Y-enriched genomic libraries.

#### Y-enriched library preparation and screening

About 100 ng of Y-DNA was fragmented into small pieces by digestion with the frequent cutter restriction enzyme *Mbo*I, digestion products were cloned into the *Bam*HI site of the pBluescript vector and directly plated on LB agar plates. Five hundred randomly selected white colonies were isolated, restricted with *Pvu*II, electrophoresed on agarose gels and sandwich transferred onto replicate nylon membranes. Membranes were pre-hybridized in the presence of high excess female genomic DNA in order to suppress female-specific signals and signals from highly repetitive elements: 70 μg of female gDNA was added, corresponding to an approximate 1000-fold excess with regard to the radioactive probe. Each set of replicate membranes was subsequently hybridized with a radioactively labeled probe from whole genomic male and whole genomic female DNA, respectively, also in the presence of 1000-fold excess female gDNA. Seventeen clones whose hybridization signal with the male probe was considerably more intense than that with the female probe were considered for further analysis. Of them, clone pFF5 is analyzed in this publication.

### Library screening, subcloning and sequencing

Preselected library fractions of an adult olive fly library in λ DASH II [41,42] was screened with a probe containing an amplified *Achilles*-fragment. This PCR fragment (Achill400), targeting part of the *gag* gene of *Achilles* (S1 Table), was 338 bp in length and further labeled with biotin-11-dUTP using a random primer DNA labeling kit (Fermentas, Burlington, Canada). Positive phage plaques were selected and rescreened. Four positive phage clones were isolated using standard protocols [43] and analyzed by restriction mapping using the restriction endonucleases *Eco*RI and *Hind*III. The clone Φ443, which had the largest insert, was selected for further analysis. Approximately 2.5 μg of phage DNA was digested by the enzymes *Eco*RI and *Hind*III which generate 0.9, 1.9, 1.35, 4,8 and 6.0-kb-long fragments, separated on 1% agarose gels and transferred onto Hybond-N+ nylon membranes (Amersham Biosciences) using neutral transfer. Southern hybridization was performed according to standard protocols described by [43] at 60°C using 20 ng/ml of the biotin-labeled probe Achill400. DNA restriction fragments of the lambda clone Φ443were subcloned into pBluescript SKII+ and then sequenced by Macrogen Inc (Korea). DNA sequences were assembled using Omiga (International Biotech Inc., CT) and searched for homologies using BLAST [44] programs. To ensure that no small fragments were missing from the junctions of the subclones p443-0.9E and p443-1.9H and subclonesp443-1.9H and p443-1.35EH, the amplified in-between regions based on the flanking sequences (S1 Table), were further cloned and sequenced.

An approximately 6.0 kb region upstream of the subcloned fragments was amplified by long PCR and its sequence was determined by primer walking (S1 Fig). After an initial round of sequencing from a known sequence at one end of the template, each subsequent round was initiated from a new primer, which was based on the end of the sequence obtained from the previous reaction (S2 Table).

The 3’ coding sequence as well as its downstream region that was missing from the analyzed phage clone were determined using a modified protocol of Pearce et al.[45] for the rapid isolation of LTR sequences (S2 Fig). Genomic DNA was partially digested with *Taq*I and the appropriate adapters (Taq1 adapter 5’-GACGATGGATCCTGAG, Taq2 adapter 5’-CGCTCAGGATCCAT) were ligated according to standard procedures [46]. Subsequently two semi-nested PCRs were performed, with nested primers specific to *Achilles* sequences and to the adapters (S3 Table), prior to subcloning and sequence determination.

### Chromosome preparations and *in situ* hybridization

Spread preparations of mitotic and polytene chromosomes were made from the brain (cerebral ganglia) and the salivary glands, respectively, of third instar larvae and young pupae (1–2 days old) [34]. Probes were labeled with digoxigenated dUTP (Dig-11dUTP) using the random priming method. Hybridization was performed at 62°C and signals were detected with specific antibodies (ROCHE Diagnostics, Mannheim, Germany) according to [47]. Detailed description of the pretreatment of chromosome preparations, hybridization, detection and image analysis are presented in [31,48].

### RNA isolation and qRT-PCR expression analysis

For the RNA extractions were used: 1) two virgin female and two virgin male (i.e., two biological replicates per sex) adult *B*. *oleae* flies from the “Demokritos” laboratory strain, 2) two individual eggs (two biological replicates) from the various time points during the embryonic developmental stages (as described in [49]), 3) two pools of five pairs of testes and two pools of five ovaries (two biological replicates per tissue) before mating. The total RNA of each sample was isolated using TRIsure-reagent (Bioline, London, UK) according to the manufacturer's instructions and subsequently treated with TURBO DNA-free DNase (Ambion®, USA) to remove any residual DNA contamination. 1 μg of total RNA was used as a template for random priming reverse transcriptions (RT) with the MMLV Reverse Transcriptase (GeneON, Germany). Reverse transcription was conducted at 42°C for 50 min and 70°C for 15 min. Standard amplifications of a 0.4 kb intron crossing fragment of BoEST_175 were carried out to test for DNA contamination using the primers epic175F and epic175R (S1 Table,[50]).

One-tenth of the resulting cDNA was further used to validate the expression profiles by a relative quantitative Real time-PCR approach, with primers specific for the reverse transcriptase region (RT) of *Achilles* (S1 Table). qPCR reactions were carried out in a total volume of 20 μl consisting of 1μl of template DNA, 1x of the iTaq Universal SYBR Green Supermix (Biorad, Gaithesburg, MD) and 400nM of each primer (S1 Table). The thermal cycling conditions were: 95°C 10 min, 95°C 10 s, 53°C 10 s, 72°C 10 s for 40 cycles. qPCR was carried out on CFX Connect Real-Time PCR Detection System and data analyzed using the CFX Manager™ software. No template control (NTC) was also included in each experimental run as negative control to verify that no reagent contamination had occurred by the target DNA. Triplicate reactions were conducted in each assay and expression values were calculated relatively to the housekeeping *rpl19* gene.

### TE copy number estimation

To evaluate the copy number of the element in the olive fly genome, an approach based on absolute Real-time qPCR using SYBR Green I dye was performed as described in detail previously [51,52]. The absolute quantity of the element in the genomic DNA is obtained by interpolating the Ct value of the target sequence (*Achilles*-RT) against the standard curve generated by the dilution series of a standard plasmid. Each PCR reaction was performed using as template either the cloned RT (standards) or genomic DNA (unknowns). Initially, a series of the 1.350 kb *Eco*RI/*Hind*III fragment containing the *Achilles*-RT dilutions were prepared (1.071 fg, 21.437 fg, 8.575 pg, 171.5 pg) and used as an external standard for PCR. The fragment was gel isolated by digesting the recombinant plasmid p443-1.35EH with the respective enzymes. The starting copy number of the standard in each dilution was obtained based on mass, concentration and size parameters according to the equation[53]:

$$DNA\;(copy)=\frac{6.22\times 10^{23}\left(copies/mol\right)\times DNA\;mass\left(gr\right)}{DNA\;size\left(bp\right)\times 660\left(gr/mol\right)}$$

Subsequently, the Ct values measured by the qPCR for each standard dilution automatically generated the standard curve (measured Ct values against *Achilles*-RT copies). The genomic samples were determined by three replicates in each experiment. Finally, the *Achilles* copies in the unknown male or female genomic DNA sample (0.75 ng per reaction) were determined by interpolating its Ct value against the logarithm of their initial template copy numbers of the standard curve. Both the standard plasmid and the unknown DNA were PCR amplified with the same primer set under identical reaction conditions.

### Sequence alignments

To explore the phylogenetic relationship of *Achilles* with other elements of the *BEL/Pao* family, the reverse transcriptase (RT) amino acid sequences were used in multiple sequence alignments with CLUSTALX [54]. Phylogenetic trees were constructed on the basis of the neighbor-joining method using MEGA v.2.1 program [55] using the neighbor-joining method. Bootstrap values were estimated from 1000 pseudoreplicates.

## Results

### Screening of a Y-enriched library and isolation of a retrotransposon related sequence

**Fig 1:**
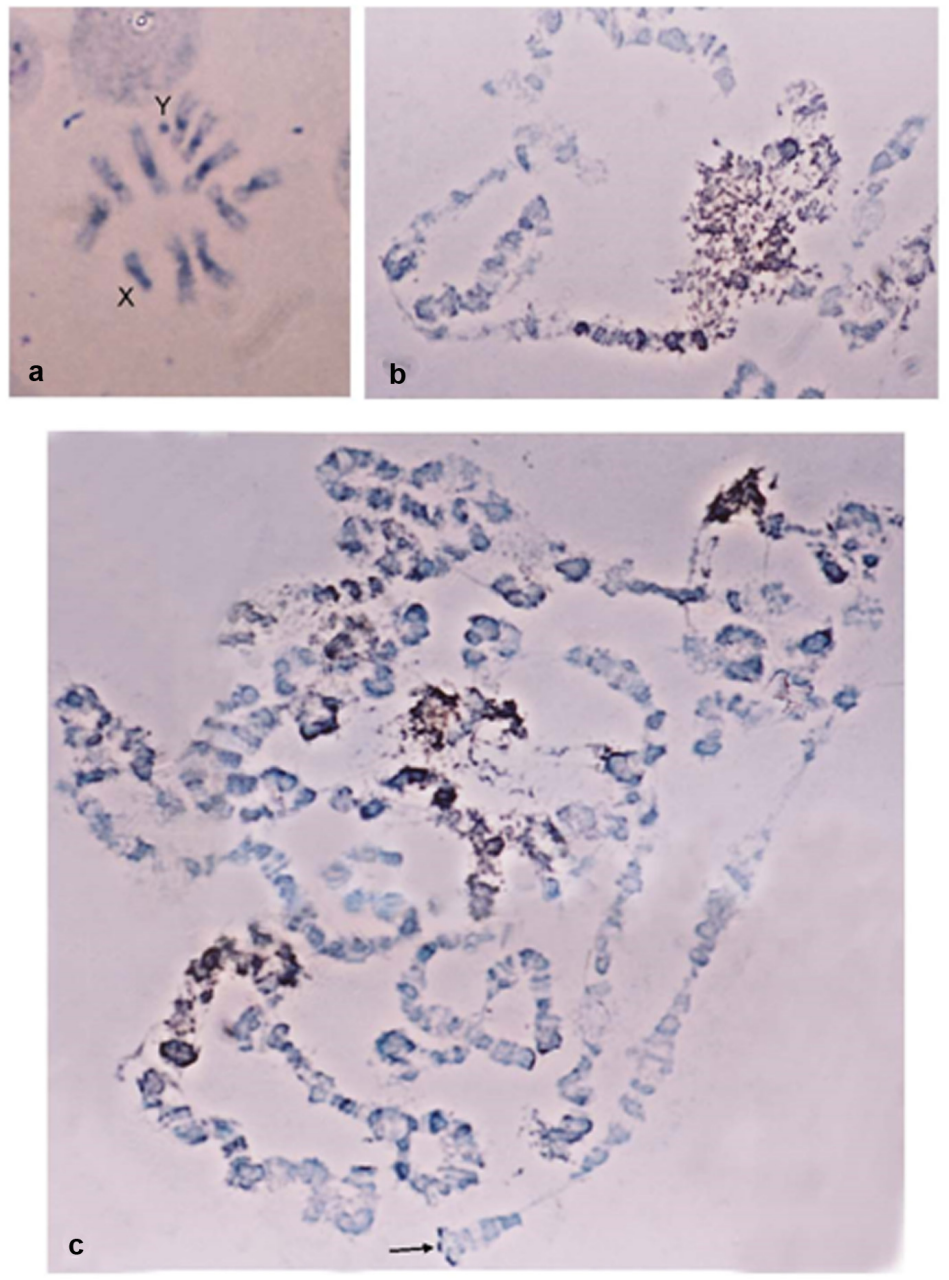
Cytogenetic distribution of partially Y-enriched libraries with preference towards the male DNA. *In situ* hybridization to *B*. *oleae* a) metaphase & b, c) polytene chromosomes revealed that the element is dispersed throughout the genome and also located at heterochromatic regions.

The particularly small size of *B*. *oleae* Y chromosome, as this is established by the fly’s karyotypic analysis [56], enabled the isolation of a ~4 Mb zone in a PFGE, presumably belonging to the Y chromosome. In order to verify the origin of the isolated material, it was further used as a probe in *in situ* hybridization on metaphase and polytene chromosome preparations (Fig 1). Hybridization signal was detected on the entire Y chromosome, as well as ona large part of the X chromosomes and the centromeric regions of all autosomes (Fig 1a). On the polytene spreads hybridization was observed on the heterochromatic network corresponding to the sex chromosomes, as well as on the centromeric regions and dispersed bands of the polytene element (Fig 1b and 1c).These results demonstrate that the PFGE isolated material included sequences preferentially located on the Y chromosome and on heterochromatic regions throughout the chromosomes. Most likely, these sequences are repetitive elements known to accumulate in heterochromatic and peri-centromeric regions.

Subsequently, this material was used to prepare Y-enriched libraries. Screening of these Y libraries with male and female genomic DNA as probes yielded several clones with differential male preference. One of these clones, pFF5, showed a strong hybridization signal with male DNA compared to female genomic DNA (S3 Fig). Based on this pattern of preferential hybridization in male DNA, together with the fact that this clone was identified using a Y-enriched probe, we speculated that this might represent a repeats equence on the Y chromosome. Sequence analysis indicated a *gag*-encoding region with identity to Pao-like retrotransposons [57].

In order to isolate full-length copies of the putative retrotransposon, a PCR product of clone pFF5 was used as a probe to screen a λDASHII *B*. *oleae* genomic library. Four putative retrotransposon-containing lambda phage clones were identified and mapped using restriction enzymes and analyzed by Southern blotting. Phage Φ443 that contained the largest retrotransposon sequence was further analyzed extensively. Sequencing of Φ443 subclones revealed the coding region of the putative retrotransposon, while a contiguous DNA sequence corresponding to the 5’ region of the element was obtained by primer-walking.

### Structural organization of *Achilles* and its position within the phylogeny of LTR retrotransposons

The partial genomic sequence of the novel *B*. *oleae* retrotransposon characterized here was comprised of 7,487 bp and was isolated from a phage clone that did not contain the intact element. In particular, the 3’ end of the ORF as well as the 3’ LTR sequence were absent. The 5’LTR sequence was determined to be 672 bp long, flanked by the dinucleotides TG and CA found at its ends. Downstream of this putative 5’LTR region, a primer binding site (PBS-like) sequence was identified (S4 Fig) which shared significant homology to the *D*. *melanogaster* Tyr-tRNA gene, showing complementarity to 18 nucleotides of the 3’ end of the respective tRNA that could be used to prime the element’s minus strand replication. The typical LTR poly-A transcription termination signal AATAAA was not detected. However, an ATTAAA motif was detected between 285 and 290 bp that could be used alternatively as a polyadenylation signal [58]. Direct and indirect repeats forming a complex structure were also identified within the 5’ UTR spanning the region between the LTR and the ORF (S5 Fig). Similar structures are common within the *BEL/Pao* as well as the *Gypsy* family [57,59]. One large open reading frame (ORF) was detected between positions 2588 bp and 7486 bp of the total sequence. This *in silico*-identified ORF, encodes a Gag-Pol precursor of 1632 amino acids, which is then cleaved to the respective proteins without an intervening termination codon. No envelope protein ORF was detected within the analyzed sequence. The 5’ to 3’ order of the domains in the polymerase ORF that encode for the different proteins indicated that the novel retrotransposon could be classified as an LTR retrotransposon following the arrangement of the *BEL/Pao* superfamily [59]. Nonetheless, the maximum similarity with the other members of the *BEL/Pao* superfamily is at a maximum of 49% (amino acid level, reverse transcriptase [RT] putative protein). According to Wicker et al [18], a similarity level below 80% would justify a separate family of elements within a defined superfamily. Therefore, the novel *B*. *oleae* element should constitute a new family of *BEL/Pao* LTR retroelements, that we denominate BEL-*Achilles*_Bo. The accession number given to this new element is KT280063. Therefore, based on standard nomenclature rules, the element’s name should be RLB_*Achilles*_KT280063 (denoting Retroelement Class I, LTR order, *BEL-Pao* superfamily, *Achilles* family, and its accession number). For the sake of simplicity, henceforth the element will be referred to as *Achilles*.

#### Common structural features

**Fig 2:**
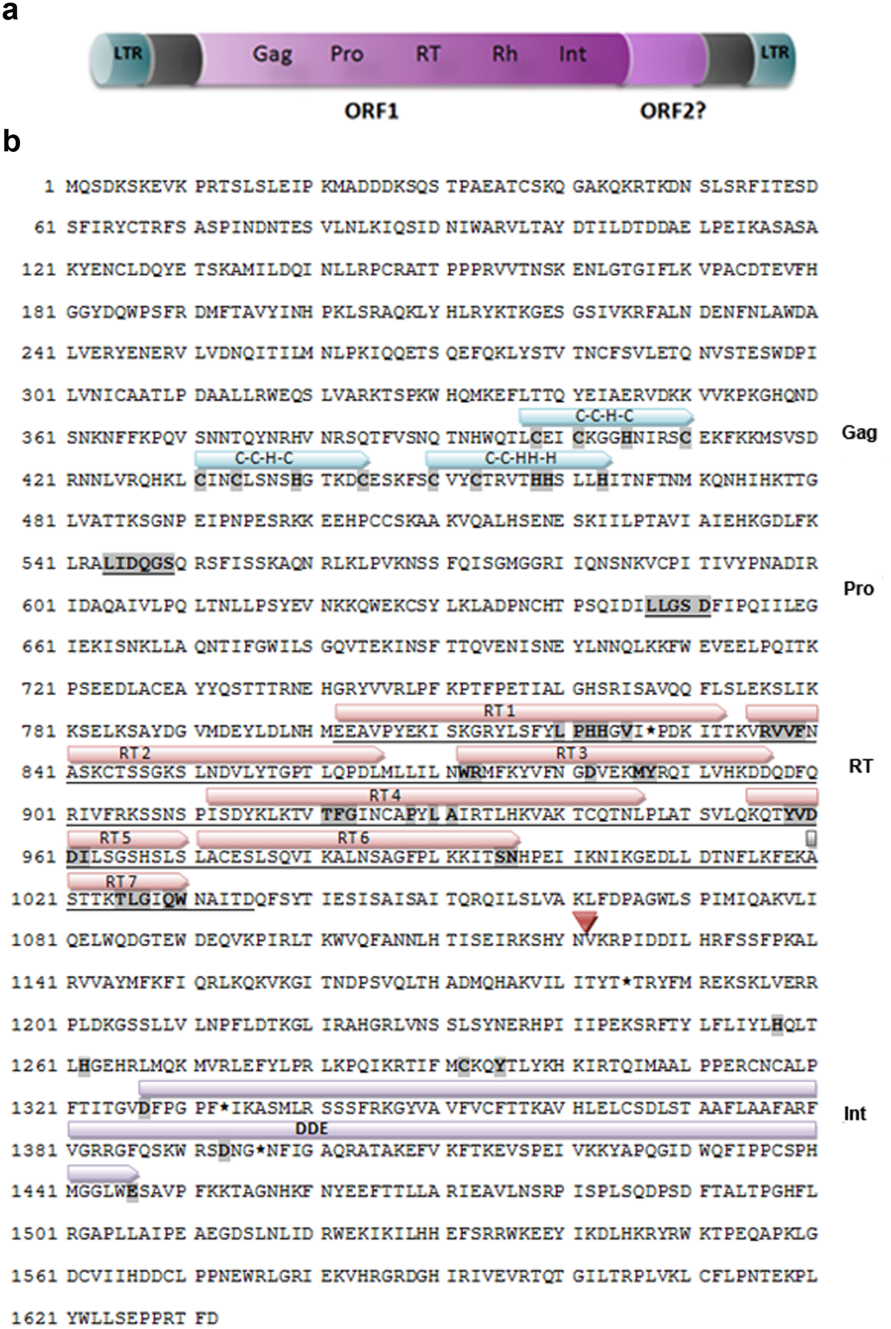
Organization and structure of the putative intact *Achilles* element. a) ORF1 encodes a Gag-Pol polyprotein consisting of 1632 aa. b) Conceptual translation of the *Achilles* coding region. The highly conserved amino acids are denoted by bold letters and highlighted in grey. The underlined amino acids and/or the above of them arrows represent the identifiable motifs shared with other retrotransposons. Their designations are shown on the right. The position marked with the red triangle (▽) indicates the deletion of the RH region. Asterisks (*) refer to the stop mutations within the coding sequences.

Two cysteine and histidine based motifs characteristic of a putative zinc-finger RNA-binding site, C-X_2-_C-X_4_-H-X-_4_-C (CCHC) [60], which are found in many LTR retrotransposons were identified in the GAG protein. Additionally, a third Cys motif C-X_2-4_-C-X_3-4_-HH-X_3-4_-H (CCHHH), which is proposed to be a specific Pao-like feature [61], was also detected. The *pol* gene domains are localized downstream of the *gag* gene, including the enzymatic domains of protease, reverse transcriptase and integrase (Fig 2).

The PRO domain encoding a potential aspartic acid protease was recognized by the typical conserved amino acid residues “LIDQGS” and “LLGSD” [62]. Following in order, the seven characteristic motifs of the reverse transcriptase (RT) were detected, as indicated in Fig 2b. Immediately downstream of the RT domain, a possible deletion was identified and the RNAse H (RH) motifs could not be found. Finally, the integrase activity domain (INT) was successfully defined. At its amino end, INT contains the conserved residues of the zinc-finger binding motif H-X_4_-H-X_29_-C-X_2_-C which is implicated in the recognition of the LTR sequences. In general, its central catalytic domain is composed of invariant aspartate (D) and glutamate (E) residues separated by 35 amino acids [17]. *Achilles*’ INT contains 52 residues between D and E, which is within the reported range of the internal residues in *Pao*-like elements [61].

Several stop codon mutations were found within the coding region, which disrupt the reverse transcriptase domain. In addition, there were missing parts from the RNaseH domain that could not be identified somewhere else within the sequence as a consequence of an internal rearrangement, suggesting its deletion. In order to test if this internal deletion is attributed only to the deduced sequence from Φ443, flanking primers to the deleted RH region were used for PCR amplification on genomic and phage DNA, respectively. The differences in length between the derived PCR products were indicative of the presence of RH in an intact element somewhere else in the *B*. *oleae* genome (S6 Fig).

**Fig 3:**
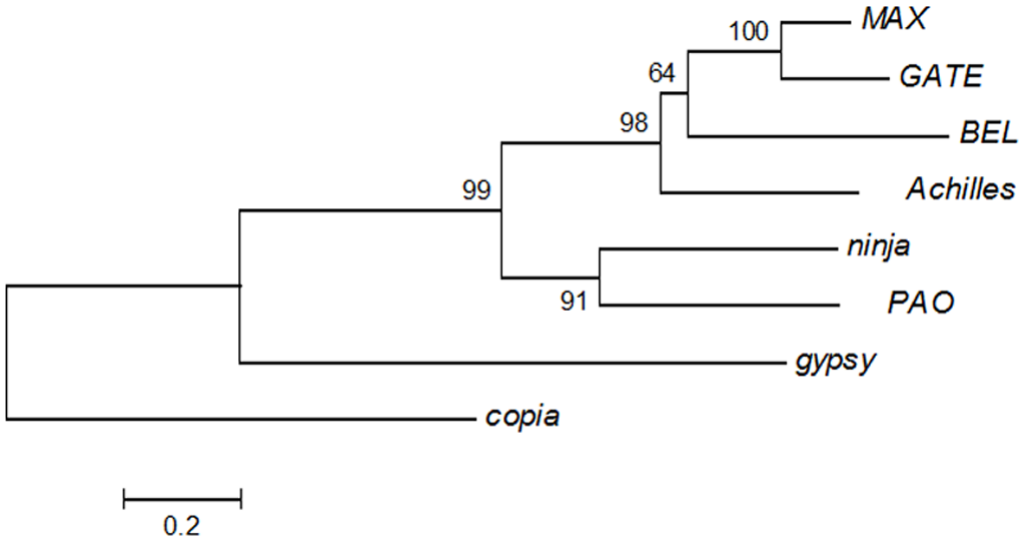
Phylogenetic analysis of *Achilles*. The RT domains among representative members of the *BEL/Pao* family (MAX, GATE, BEL, Pao, ninja) were aligned with the corresponding region of *Achilles* using the ClustalW. The N-J tree was generated using as outgroup the copia element. *Achilles* is more related to the *BEL* elements. The numbers at the nodes are the bootstrap values based on 1000 pseudoreplications.

The comparison of the amino acid sequences coding for the RT domain has been widely used to generate phylogenetic relationships and to infer the classification of retrotransposons [63]. Therefore, a multiple sequence alignment of the RT domain was conducted in order to examine the relationship of *Achilles* to five representatives from the *BEL/Pao* family (*MAX*, *GATE*, *BEL*, *Pao* and *ninja*). The constructed phylogenetic tree placed *Achilles* within the *BEL/Pao* family of LTR retrotransposons (Fig 3) and revealed that its closest relative was the *BEL* element. *Achilles* also grouped closely with *Pao* and *ninja*, whereas it is clearly distinct from the *copia*-like retrotransposon, which served as an outgroup.

### Genomic distribution of *Achilles*

**Fig 4:**
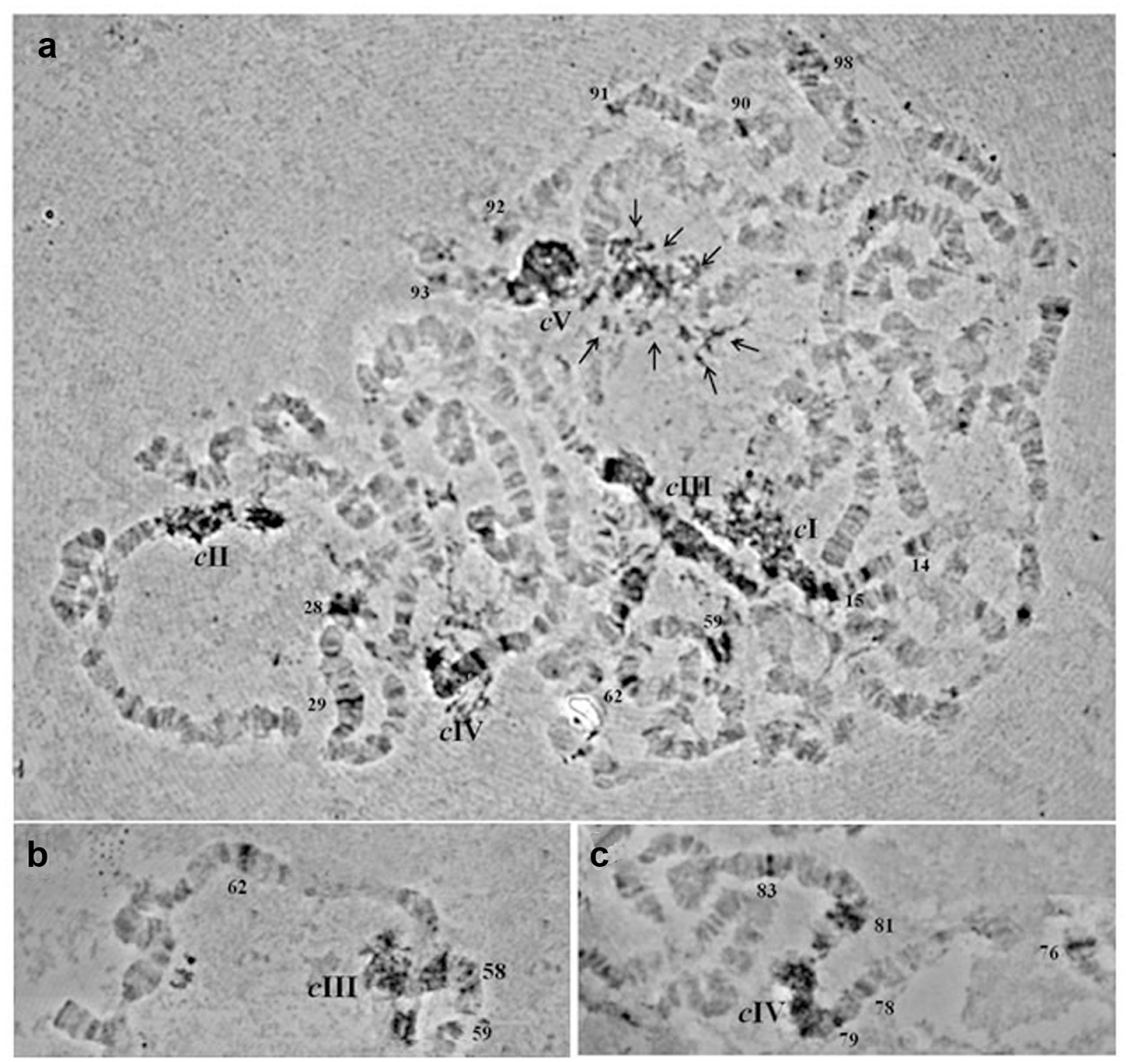
*In situ* hybridization of a digoxigenin-labelled *Achilles*-probe on *B*. *oleae* polytene chromosomes. a) Polytene nucleus, b) part of polytene chromosome III, c) part of polytene chromosome IV. Spread preparations of polytene chromosomes were made from the salivary glands of third instar larvae as described by Drosopoulou et al. (1995). Centromeres are indicated by “c” and the corresponding chromosomes are demonstrated by their number (I-V). Multiple heterochromatic signals are indicated by multiple arrows (in Panel a). Discrete euchromatic signals are indicated by the number of the respective chromosomal division (Panels a, b, c).

In order to study the chromosomal localization of the element, a digoxygenin-labelled probe of the *gag* gene was used as probe. The *in situ* hybridization of the *Achilles*-fragment on the polytene chromosome preparations showed a dispersed hybridization pattern. Multiple hybridization signals were identified in the centromeric regions of all five autosomes and in the granular heterochromatic network representing the under-replicated sex chromosomes. Moreover, about twenty two discrete bands dispersed on all polytene elements were identified as well (Table 1 and Fig 4).

**Table 1:** Cytogenetic sites of *Achilles* as revealed by *in situ* hybridization on *B*. *oleae* polytene chromosomes.

Chromosome	I	II	III	IV	V
Hybridization sites	Centromere	Centromere	Centromere	Centromere	Centromere
	IL 14	IIL 28	IIIL 58—three bands	IVL 76	VL 90
	IL 15—two bands	IIL 29	IIIR 59—two bands	IVL 78	VL 91
			IIIR 62	IVL 79	VL 92
				IVR 81—two bands	VL 93
				IVR 83	VR 98

Additionally, the exact number of *Achilles*-copies per haploid genome was evaluated by absolute qRT-PCR, comparing the measurements of a target region with a reference plasmid copy number containing the region of interest (S7 Fig). It was indicated that approximately 30–40 copies of *Achilles* reside in the genome (Table 2). Estimated copy numbers for other *BEL/Pao* retrotransposons (see [20]) demonstrate that *Achilles* is not a high copy number element. This intermediate frequency in eukaryotic genomes is characteristic of *BEL/Pao*-like elements, compared to the typically more abundant *Gypsy* elements and the low copy number *Copia* elements [19,64]. The between sexes comparison revealed a variation in *Achilles* copy number, with male flies possessing 5–10 copies more than female (CI range: 18–38 and 12–33 copies, respectively).

**Table 2:** Analysis data to estimate *Achilles* copy number by qPCR using a reference standard curve.

	Sample concentration (ng) ^a^	Calculated copy number ^b^	Copy number per haploid genome ^c^	CI for mean
**Male**	0.75	5.93E+04	27.82 ± 3.72 ^d^	12–33
**Female**	0.75	4.75E+04	23.02 ± 4.33 ^d^	18–37

^a^ Initial template concentration of the *B*. *oleae* genomic DNA (ng) used at the qPCR reactions.

^b^ Mean copy number of *Achilles* which was estimated for the initial template copies of the genomic DNA (a) based on the standard curve.

^c^ *B*. *oleae* haploid genome size: 0.352 pg

^d^ Standard Error (SE) for the triplicate measurements (n = 3)

### Species distribution of *Achilles*

**Fig 5:**
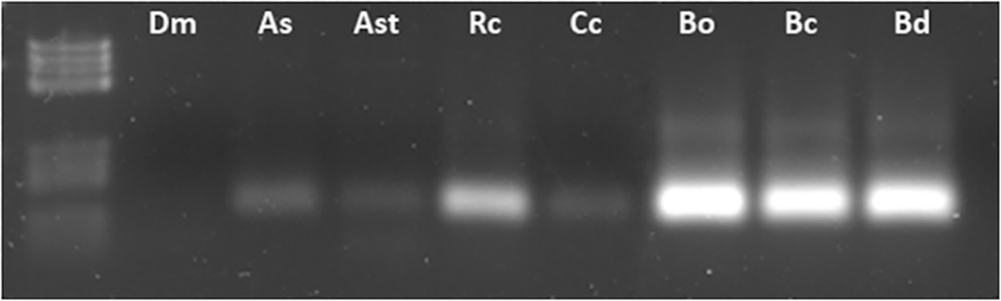
Species distribution of *Achilles*. Genomic PCR amplfication of *Achilles*-RT in in the 7 diptera species tested using AchillF2/R2 primer set. [Dm: *Drosophila melanogaster*, As: *Anastrepha serpentina*, Ast: *Anastrepha striata*, Cc: *Ceratitis capitata*, Bo: *Bactrocera oleae*, Bc: *Bactrocera correcta* and Bd: *Bactrocera dorsalis*.]

Homology tBLASTn searches with *Achilles* as a query yielded homologous sequences of *Ceratitis capitata* and *Bactrocera dorsalis*. The above result indicated that this element may be present in other Tephritidae species as well. Several representatives of the Tephritidae family were used to explore the host range of *Achilles*, including *Anastrepha serpentina* and *An*. *striata*, *C*. *capitata*, *Bactrocera oleae*, *B*. *correcta* and *B*. *dorsalis*. The *D*. *melanogaster* was also included in the analysis due to *Achilles* homology with *MAX*. Genomic amplification of the RT domain in the 7 species tested confirmed its presence within the examined genomes (Fig 5). These data suggest that this element might have been present in their common ancestor. However, the lack of amplification in evolutionarily more distant Dipteran species, such as *D*. *melanogaster*, does not exclude the presence of the element in these genomes too; rather, this may be a result of insufficient hybridization of the primers used during the PCR amplification due to sequence divergence. Nevertheless, data of *Achilles* distribution within related genomes are preliminary and there is need to investigate the intra-family relations more thoroughly to assume its evolutionary fate.

### Transcriptional activity of *Achilles*

**Fig 6:**
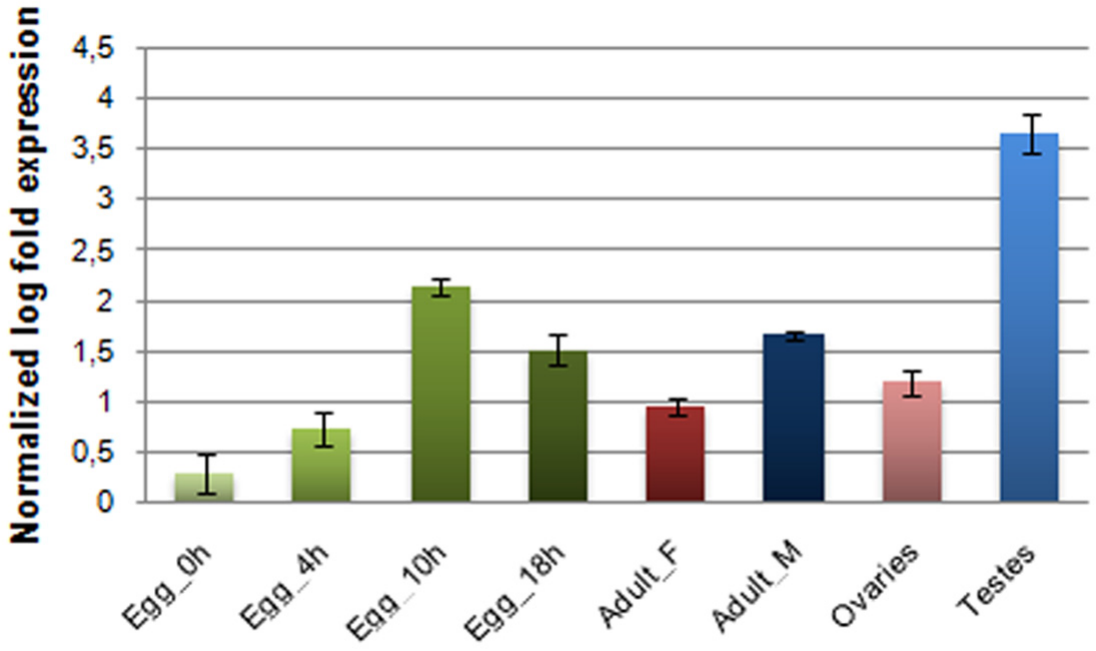
Expression profile of *Achilles* in specific tissues of both sexes as well as in embryos at various developmental stages. Expression levels were detected by relative qRT-PCR in a) individual eggs collected at different time points during embryonic development and b) in two different tissues per gender of 7^th^ day adult virgin flies: total bodies of females (Adult_F) and males (Adults_M) and the germline tissues testes and ovaries. Standard error of the mean of two biological replicates per time point is depicted in bars.

In order to assess potential transcriptional activity of *Achilles*, a relative quantitative RT-PCR was performed with specific primers. qRT-PCR products were detected in both male and female samples, demonstrating a differential level of expression between sexes (Fig 6). The detected transcriptional activity should result from an intact copy and not the impaired copy under study. Interestingly, the BLAST hits generated by tBLASTn homology searches of *Achilles* corresponded to sequences generated by RNAseq or published EST datasets, thus to active elements. For example, similarity was obtained to a male *C*. *capitata* EST dataset (JK832499.1, e-value: 9e^-43^), which is in accordance to our results.

Besides the entire insect body, germline tissues (male testes and female ovaries) as well as embryos at various developmental stages were examined for transcriptional activity. Although transcription was confirmed among the samples tested, *Achilles* expression levels showed considerable variability (Fig 6). The highest expression was observed in testes. In all cases, however, preferential male expression was affirmed. Similar observations were reported by [65] regarding a potentially active heterochromatic copy of the *GATE* element whose transcription was suggested to be regulated via an RNAi mechanism in testes.

## Discussion

Molecular and genetic studies, combined with new information from whole transcriptome analyses, have given new perspectives towards the understanding of genome structure of the olive fruit fly *B*. *oleae* ([49,66] and references therein).

*Achilles* is the first characterized retrotransposon in the *B*. *oleae* genome. Partial sequence of this novel element was first identified in a library enriched with material derived from the Y chromosome of the olive fly (Fig 1). Initially a differential hybridization approach was implemented, using as probes labeled total DNA from both sexes, in order to screen for clones preferentially hybridizing to the male probe. Based on the kinetics and dynamics of DNA hybridization of a total genome-derived probe, only highly or moderately repetitive DNA sequences would hybridize. Thus, this approach targeted male-specific repetitive DNA or sequences that are highly amplified on the Y chromosome but may be present elsewhere in the genome as well. The overall premise was that the analysis of the neighborhood around such sequences could shed light on the structural organization of the olive fly Y chromosome.

Indeed, during the analysis of the lambda phage clones that contained the putative repetitive element, we ran across sequences such as a *B*. *oleae* specific centromeric satellite repeat, BoR300 [52]. Wong and Choo [67] argued against the hypothesis that retrotransposons may be a common source for centromeric DNA by providing the evolutionary templates on which tandem repeats have evolved. In fact, apart from the presence of an *Achilles*-fragment between the repeat arrays along the heterochromatic centromeres, we have no further data to claim any association of *Achilles* with the BoR300 repeats.

The novel retrotransposon shares sequence homology and structural features characteristic of the LTR-retrotransposons of the *BEL/Pao* superfamily. In contrast to the indiscernible similarities at the nucleotide level between *Achilles* and other *BEL/Pao* elements, the conservation at the amino acid level enabled further molecular characterization. The different conservation between the two levels can be a consequence of diverse codon usage pattern of each species [50,68] that is probably influencing the evolutionary rate of such elements.

*Achilles* displayed an RNA binding motif characteristic of *gag BEL/Pao* elements followed by domains of enzymatic action protease, reverse transcriptase (RT), RNAseH, and integrase, in that order (Fig 2). It was also found to be related to other discrete families but tends to cluster with *BEL* retrotransposons. Notwithstanding such similarities, *Achilles*’ differentiation from its closest relatives at the nucleotide and protein level would define a separate family of retrotransposons within the BEL/Pao superfamily.The particular element that we analyzed in detail seemed to be defective. However, future genome sequencing will allow an assembled consensus sequence of the likely active element, as well as the discovery of other members of the *Achilles* family.

The *Achilles* copy isolated from phage clone Φ443 is clearly inactive due to accumulated stop mutations. However, a qRT-PCR approach demonstrated the presence of *Achilles* transcripts. While this finding indicates potential mobility of *Achilles* at this point in evolutionary time, further studies need to be conducted in order to explore this scenario. In order to explore the transcriptional activity in diverse developmental stages of *B*. *oleae*, *Achilles* transcripts were sought in embryonic through adult tissues. Indeed, different levels of expression were observed in the various tissues tested, with the germline tissues of males possessing the most abundant transcripts (Fig 6). The functional role of the detected transcriptional activity during embryogenesis is still an open issue and may be indicative of a probable involvement of *Achilles* in cell differentiation and organogenesis. The exact role of retrotransposon transcripts in gonadal somatic cells is still obscure. Clearly, however, the expression and retrotransposition in germ line would be a more effective strategy for retrotransposons to ensure their inheritance to the next generation. In that regard, retrotransposons may use this path to obtain additional access to gametes, as somatic expression may detour the host defense against retrotransposons in the germ line. The reported transcript abundance of LTR retrotransposons and RNAi-related genes in the gonadal somatic cells of *D*. *melanogaster* embryos, led to the hypothesis that the transcripts are inactivated by a post-transcriptional regulatory mechanism [69]. The same authors proposed that LTR-retrotransposon transcripts would act as a “trigger” for their subsequent processing by RNAi pathway to produce siRNA, which in turn silences the retrotransposons in the following developmental stages.

The proportion of genome occupied by *Achilles* was estimated by a qPCR approach, which revealed a variation in *Achilles* copy number between sexes. The extra male copies might correspond to Y-specific integrations. We consider that the calculated copy number range is in reasonable agreement with the *in situ* hybridization results, which allowed the physical mapping of the retrotransposon on *B*. *oleae* polytene chromosomes. The *in situ* hybridization data reveal an interspersion pattern of the element throughout the insect’s polytene chromosomes (Fig 4 and Table 1). *Achilles* is concentrated at non-polytenized heterochromatic regions but some copies were found to be located on discrete bands of polytenized elements as well. Similar distribution was also observed in *D*. *melanogaster MAX* element mapping on polytene as well as on mitotic chromosomes [57].

Hybridization signals of *Achilles* on these regions strongly suggest that these chromosomes may either act as a refuge or a trap for *Achilles* copies. Since in heterochromatic and pericentromeric regions recombination is suppressed and there is no selection against insertional mutations. Therefore, the accumulation of a considerable amount of copies in such genetically inert areas could be indicative of their reduced impact on genes or genome. Thus, it represents another example demonstrating that these genomic regions comprise favorable refuges for retroelements [45].

In addition, there is evidence that the presence of transposons themselves can contribute to the formation of heterochromatin. For example, there is compelling evidence that the accumulation of TEs, especially retrotransposons, triggered Y chromosome degeneration in *D*. *miranda* [27]. Several molecular studies in different taxa demonstrated that during early evolution of the chromosomes, a sequence reshaping within new sex chromosome regions is occurring through modifications of the chromatin structure and the insertion of repetitive DNA sequences [23]. These morphologically and genetically induced alterations together with the absence of recombination favor the differentiation of sex chromosomes and lead to genetic erosion of the Y chromosome. Sex determination mechanisms, on the other hand, appear to be a labile feature and demonstrate a wide variety within Diptera [70]. Unlike *D*. *melanogaster*, in which male development is determined by the X chromosome dosage, in Tephritids sexual differentiation relies on the male-determining factor, M, which is linked to the Y chromosome [38,71,72].Interestingly, in the midge Chironomus the male sex determiner has been associated with a single heterochromatic band consisted of highly repetitive DNA and transposable elements [73,74]. Therefore, the in depth exploration of sex chromosomes structure will improve our understanding of their origin and divergence (degeneration) as well as the evolution of genetic sex determination pathways which are attributed to them. Furthermore, due to the fact that sexual differentiation and male fertility comprise a crucial part of insect control strategies, such as the sterile insect technique (SIT), the manipulation of sequences involved in these processes is of particular interest. In this context, the analysis of the Tephritidae sex chromosomes can contribute towards the establishment of new genetic tools for sex separation strategies, since only males are the active component for SIT and effective population control [75].

In this report, the analysis of *Achilles* revealed several of its features, such as its abundance and distribution in the olive fly genome or its structural plasticity. Even if the assembly of Y’s protein-coding genes is still a challenge, there are hints supporting the notion that Y heterochromatin may have functional consequences on gene regulation, raising the possibility that variation in heterochromatin, such as copy number of transposable elements and simple-sequence repeats, is under selection [76]. Therefore the unraveling of the evolutionary history of such elements which integrated into heterochromatin enriched regions, as is the Y chromosome, may ultimately improve the efforts aiming at the manipulation of otherwise intractable regions of the genome. *Achilles*-copies found on the Y chromosome are also likely to harbor complex sequences that may be informative in studies of population genetics and Y chromosome evolution. However, compelling secrets regarding to whether *Achilles* is linked to the heterochromatinization of the Y chromosome (by either genetic or epigenetic mechanisms), or to its functional and structural impact on Y-linked genomic regions (such as, for example, the maleness factor) still evade. While such questions are being considered, Achilles’ heel still remains well protected.

## Supporting Information

S1 Fig Primer walking approach based on restriction map of Φ443. a) The upstream region of 6.0 kb was initially amplified with long PCR. b) Subsequently, the sequence of the amplification product was determined by primer walking. The designed primers as well as the order of their use regarding the sequencing of the 3’ end are schematically represented on the Fig. The primers used for the sequencing of the 5’ end are reported in S2 Table.
(TIF)

S2 Fig Schematic representation of the modified protocol used for the isolation of the 3’ coding region. a) Partial digestion of genomic DNA with the restriction endonuclease *Taq*I. b) Isolation of the fragments with the desirable length and ligation of the adapters Taq-AD. c) PCR amplification using the primers 443–0.9 F1 and TaqI AP. d) Semi-nested PCR on the previously amplified region using the primers 443–0.9 F1 and TaqI AP. e) Cloning and sequencing of the amplicons.
(TIF)

S3 Fig Screening of partially Y-enriched libraries. Clone pFF5 with the highest differential hybridization to male DNA disclosed a fragment of a putative repetitive element.
(TIF)

S4 Fig Sequence and structural characteristics of the LTR and the PBS. Flanking inverted repeats of the 674 bp long 5’ LTR are shown boxed. Immediately following the 5′ LTR the putative PBS (677–690 bp) complementary to the 3′ end of tRNA-Tyr is located. A clearly identifiable putative TATA box was not found, thus not indicated.
(TIF)

S5 Fig Dot plot comparison of *Achilles* 5’ region versus itself. The analyzed region spanned the 5’ LTR (including 5’UTR) until the beginning of the *Achilles* ORF and was aligned using the program Omiga 2.0. The central black line corresponds to identical residues. Additional similarities of shorter regions (blue or red dots) are due to repeats found in LTRs.
(TIF)

S6 Fig PCR amplification of the RH region. The genomic amplification product is larger (2500 bp) than that of the phage (1900 bp), suggesting the presence of the RH region in the genomic DNA, unlike the Φ443 DNA were RH was deleted. The other bands in Lane 1 most likely correspond to non-intact *Achilles* elements in the genome. Lane 1, genomic *B*. *oleae* DNA; Lane 2, Φ443 phage DNA; Lane 3, negative control. L: molecular weight marker SM0331 (GeneON).
(TIF)

S7 Fig Estimation of copy number using the standard curve method of an external control plasmid sample. Standard curve generated from the control plasmid DNA containing RT domain of *Achilles*. Fluorescent threshold values (CT) were plotted against the logarithm of the starting copy number to produce a linear function. The slope and intercept of the curve were calculated from the linear equation describing the standard curve.For the construction of the curves, serial 10-fold dilutions of plasmid DNA were used (1.071 fg, 21.437 fg, 8.575 pg, 171.5 pg) converted to copy numbers.
(TIF)

S1 Table Primer sequences and parameters of the standard PCR (1), RT-PCR (2), absolute qPCR (3) and relative qPCR assay (4). (DOCX)

S2 Table Primer sequences used in PCR reactions to amplify the upstream 6.0 kb region (Long PCR), as well in the primer-walking approach. (DOCX)

S3 Table Primer sequences and parameter used in the initial standard PCR and the subsequent semi-nested PCR reactions to detect the downstream 3’ region of *Achilles*. (DOCX)
