# Supplementary figures and images for: Achilles, a New Family of Transcriptionally Active Retrotransposons from the Olive Fruit Fly, with Y Chromosome Preferential Distribution
Source: PLoS One. 2015 Sep 23;10(9):e0137050. doi: 10.1371/journal.pone.0137050 (PMC4580426; doi:10.1371/journal.pone.0137050)

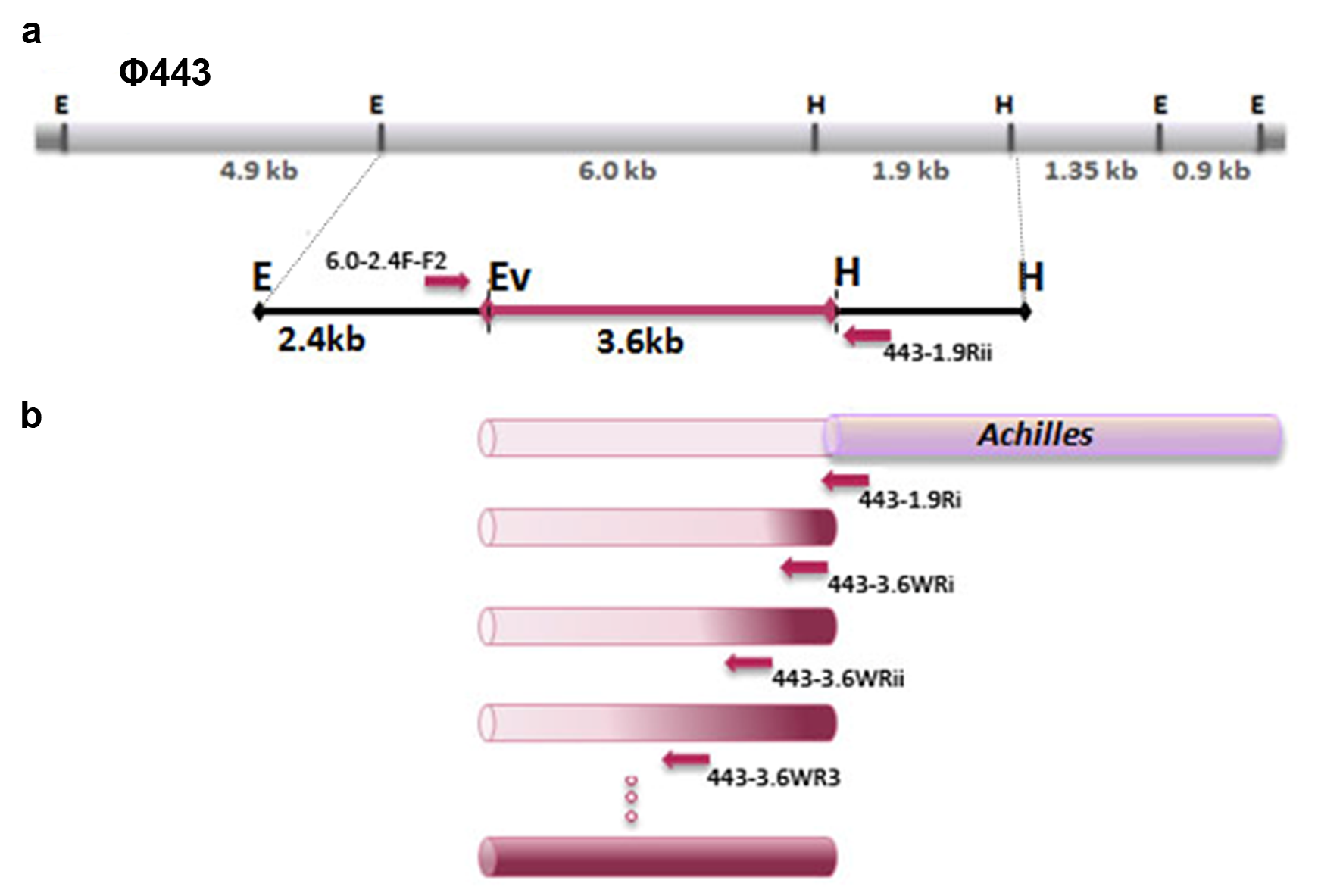

Supplement: S1 Fig — a) The upstream region of 6.0 kb was initially amplified with long PCR. b) Subsequently, the sequence of the amplification product was determined by primer walking. The designed primers as well as the order of their use regarding the sequencing of the 3’ end are schematically represented on the Fig. The primers used for the sequencing of the 5’ end are reported in S2 Table. (TIF) [file pone.0137050.s001.tif]

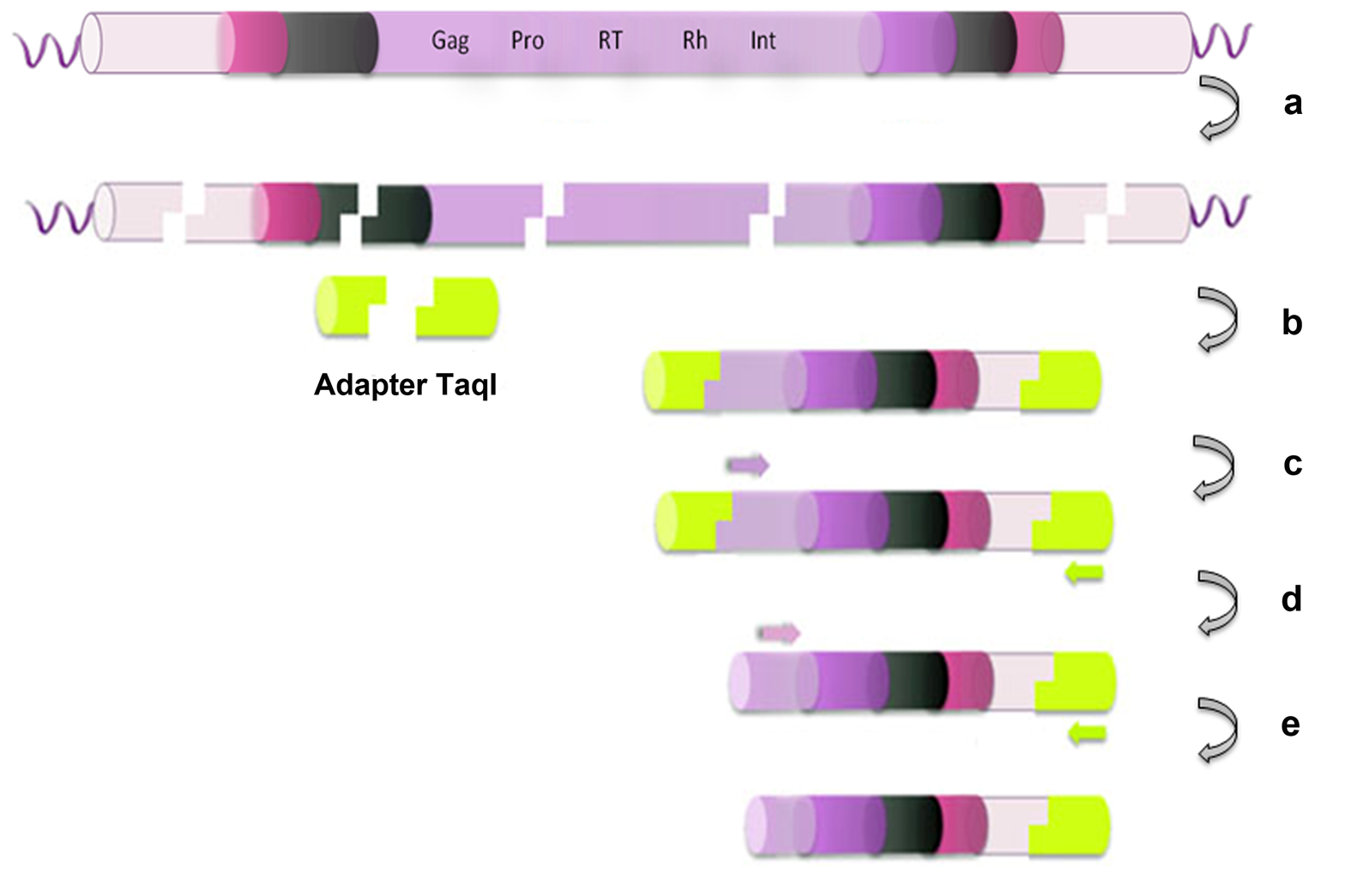

Supplement: S2 Fig — a) Partial digestion of genomic DNA with the restriction endonuclease TaqI. b) Isolation of the fragments with the desirable length and ligation of the adapters Taq-AD. c) PCR amplification using the primers 443–0.9 F1 and TaqI AP. d) Semi-nested PCR on the previously amplified region using the primers 443–0.9 F1 and TaqI AP. e) Cloning and sequencing of the amplicons. (TIF) [file pone.0137050.s002.tif]

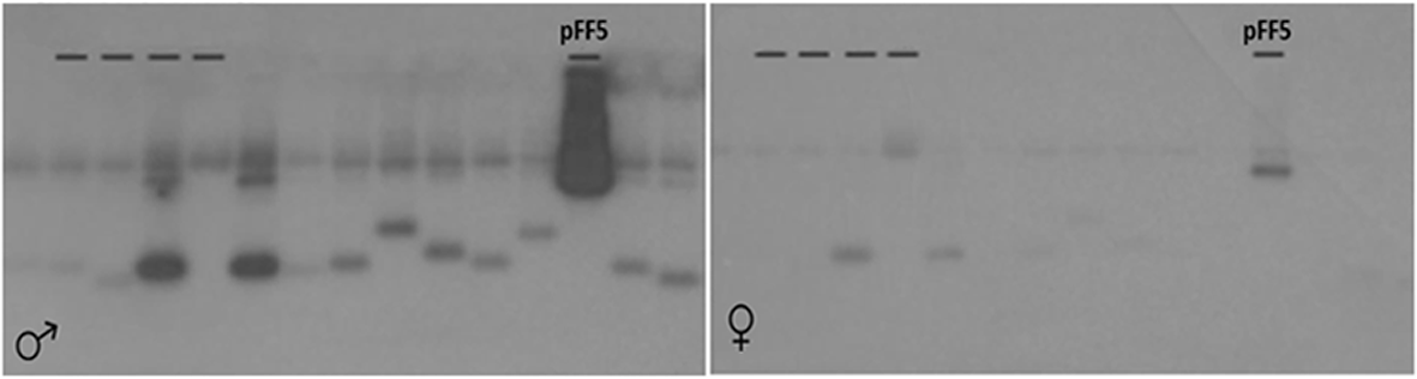

Supplement: S3 Fig — Clone pFF5 with the highest differential hybridization to male DNA disclosed a fragment of a putative repetitive element. (TIF) [file pone.0137050.s003.tif]

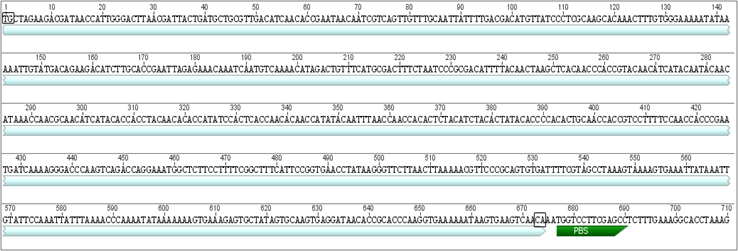

Supplement: S4 Fig — Flanking inverted repeats of the 674 bp long 5’ LTR are shown boxed. Immediately following the 5′ LTR the putative PBS (677–690 bp) complementary to the 3′ end of tRNA-Tyr is located. A clearly identifiable putative TATA box was not found, thus not indicated. (TIF) [file pone.0137050.s004.tif]

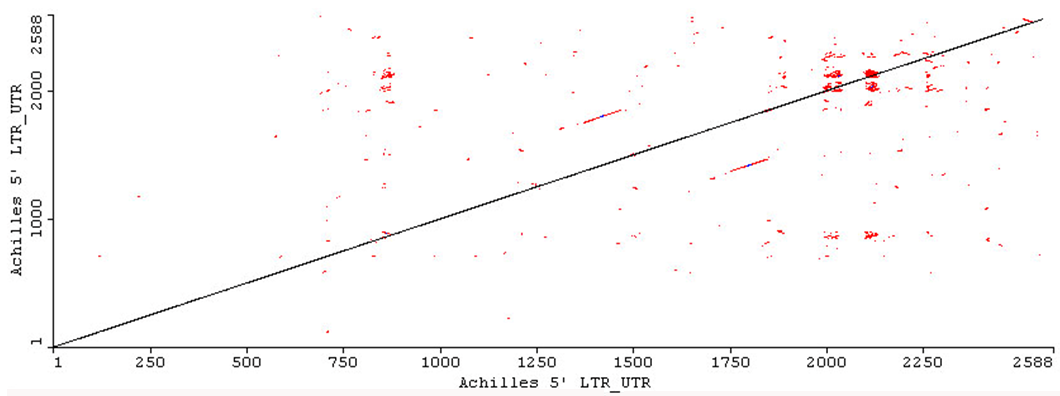

Supplement: S5 Fig — The analyzed region spanned the 5’ LTR (including 5’UTR) until the beginning of the Achilles ORF and was aligned using the program Omiga 2.0. The central black line corresponds to identical residues. Additional similarities of shorter regions (blue or red dots) are due to repeats found in LTRs. (TIF) [file pone.0137050.s005.tif]

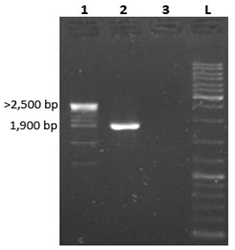

Supplement: S6 Fig — The genomic amplification product is larger (2500 bp) than that of the phage (1900 bp), suggesting the presence of the RH region in the genomic DNA, unlike the Φ443 DNA were RH was deleted. The other bands in Lane 1 most likely correspond to non-intact Achilles elements in the genome. Lane 1, genomic B. oleae DNA; Lane 2, Φ443 phage DNA; Lane 3, negative control. L: molecular weight marker SM0331 (GeneON). (TIF) [file pone.0137050.s006.tif]

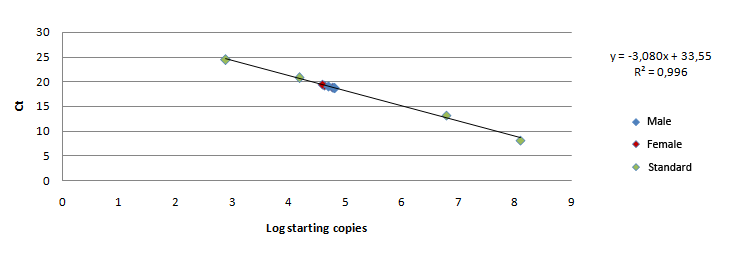

Supplement: S7 Fig — Standard curve generated from the control plasmid DNA containing RT domain of Achilles. Fluorescent threshold values (CT) were plotted against the logarithm of the starting copy number to produce a linear function. The slope and intercept of the curve were calculated from the linear equation describing the standard curve.For the construction of the curves, serial 10-fold dilutions of plasmid DNA were used (1.071 fg, 21.437 fg, 8.575 pg, 171.5 pg) converted to copy numbers. (TIF) [file pone.0137050.s007.tif]
