# Supplementary material for: Achilles, a New Family of Transcriptionally Active Retrotransposons from the Olive Fruit Fly, with Y Chromosome Preferential Distribution
Source: PLoS One. 2015 Sep 23;10(9):e0137050. doi: 10.1371/journal.pone.0137050 (PMC4580426; doi:10.1371/journal.pone.0137050)
Supplement: S1 Table — (DOCX) [file pone.0137050.s008.docx]

**S1 Table**. Primer sequences and parameters of the standard PCR (1), RT-PCR (2), absolute qPCR (3) and relative qPCR assay (4).

| Target sequence | Primer | Primer sequence (5’→ 3’) | Ta (°C) | Amplicon size (bp) | Assay |
| --- | --- | --- | --- | --- | --- |
| *Achilles*-gag | Achill400F | AGTGTTCTGATCAATGGC | 47 | 433 | 1, 2, 3 |
|  | Achill400R | CAGCATCAGGTAGTGTCG |  |  |  |
| Junction p443-1.9H/p443-1.3EH | 1.9-1.3 gapF | CTACCCCAAATCACAAAACC | 55 |  | 1 |
|  | 1.9-1.3 gapR | GTCATATTACCCCGTGATGC |  |  |  |
| Junction p443-1.3EH /p443-0.9E | 1.3-0.9 gapF | AAAGTTAGTCGAAAGACGACC | 55 |  | 1 |
|  | 1.3-0.9 gapR | TACAAAGACAGCCACATACC |  |  |  |
| BoEST_175 | epic175F | AAAATGCGCTTCCATAAGATCG | 53 | 420 | 2 |
|  | epic175F | ATCCAACATCCTTGGAATATCG |  |  |  |
| *Achilles*-RT | AchillF2 | TGATCTCATGCTGCTAATAC | 55 | 112 | 4 |
|  | AchillR2 | CTGGAAATCTTGGTCATC |  |  |  |
| rpl19 | rpl19F | GCAAGAACACAGAAGCTC | 55 | 122 | 4 |
|  | rpl19R | ACGTCTTAATACTCGTTGAC |  |  |  |
