# Supplementary material for: Achilles, a New Family of Transcriptionally Active Retrotransposons from the Olive Fruit Fly, with Y Chromosome Preferential Distribution
Source: PLoS One. 2015 Sep 23;10(9):e0137050. doi: 10.1371/journal.pone.0137050 (PMC4580426; doi:10.1371/journal.pone.0137050)
Supplement: S2 Table — (DOCX) [file pone.0137050.s009.docx]

**S2 Table**. Primer sequences used in PCR reactions to amplify the upstream 6.0 kb region (Long PCR), as well in the primer-walking approach.

| Primer | Primer sequence (5’→ 3’) | Ta (°C) | Procedure used |
| --- | --- | --- | --- |
| 443-1.9Rii | TATCTTTCGACCAGTGCATCC | 53 | Long PCR |
| 6.0-2.4F-F2 | TCAATGAACTACTACGCACAC | 53 | Long PCR |
| 443-1.9Ri | GACGGCCATTGATCATAACCTC |  | 3’ sequencing |
| 443-3.6WRi | TTGCCGAAATTGCTTACTG |  | 3’ sequencing |
| 443-3.6WRii | GCGGGAAATGTGCCAATG |  | 3’ sequencing |
| 443-3.6WR3 | CTTACCCTTTGCTTATGG |  | 3’ sequencing |
| 6.0-2.4F-F2 | TCAATGAACTACTACGCACAC |  | 5’ sequencing |
| 443-3.6WFi | GCATATTCAAAGCTAAGAC |  | 5’ sequencing |
| 443-3.6WFii | ACATCTTGCACCGAATTAGAG |  | 5’ sequencing |
| 443-3.6WF3 | CGCGAAATCAATGGATATAC |  | 5’ sequencing |
