# Supplementary material for: Achilles, a New Family of Transcriptionally Active Retrotransposons from the Olive Fruit Fly, with Y Chromosome Preferential Distribution
Source: PLoS One. 2015 Sep 23;10(9):e0137050. doi: 10.1371/journal.pone.0137050 (PMC4580426; doi:10.1371/journal.pone.0137050)
Supplement: S3 Table — (DOCX) [file pone.0137050.s010.docx]

|  | Primer | Primer sequence (5’→ 3’) | Ta (°C) | Assay |  |  |
| --- | --- | --- | --- | --- | --- | --- |
| Forward | **TaqIAP** | GATGGATCCTGAGCGA | 45 | PCR / Semi-nested PCR | | |
| Reverse | **443-0.9 F1** | TTCAAGACCCATCTCACCAC | 45 | PCR | |  |
|  | **443-0.9 F2** | GCTCCACTTCTCGCCATACC | 45 | Semi-nested PCR | |  |
|  | **L19-F1** | GACTAGGCCGCATTGAAAAGG | 45 | PCR | |  |
|  | **L19-F2** | CGAGATGGTCACATAAGAATAG | 45 | Semi-nested PCR | |  |

**S3 Table.** Primer sequences and parameter used in the initial standard PCR and the subsequent semi-nested PCR reactions to detect the downstream 3’ region of *Achilles*.
